# Suicidal ideation, attempt and associated factors among adult cancer patients at Jimma University Medical Center, Jimma, Ethiopia, 2023

**DOI:** 10.3389/fpsyt.2025.1461071

**Published:** 2025-03-13

**Authors:** Kunuya Kunno, Asmare Belete, Tamrat Anbesaw, Monenus Teshome, Shimelis Girma Kassaye, Badiru Dawud, Zelalem Birhan

**Affiliations:** ^1^ Department of Psychiatry, College of Medicine and Health Science, Wollo University, Dessie, Ethiopia; ^2^ Department of Psychiatry, Institute of Health, Faculty of Medical Sciences, Jimma University, Jimma, Ethiopia

**Keywords:** suicidal ideation, suicidal attempt, suicide, cancer, Jimma, Ethiopia

## Abstract

**Background:**

Cancer has been associated with an increased risk of suicidal behaviors and suicide has been one of the leading causes of non-cancer-related mortality among cancer patients in recent years. However, there is limited evidence on suicidal behaviors in patients diagnosed with cancer at Jimma University Medical Center.

**Objective:**

This study aimed to assess the magnitude and associated factors of suicidal ideation and attempts among cancer patients at Jimma University Medical Center, Ethiopia, in 2023.

**Methods:**

A hospital-based cross-sectional study was conducted among 271 cancer patients at Jimma University Medical Center from 1 to 30 November 2023. Data were collected using interviewer-administered questionnaires and a consecutive sampling technique was employed. Suicidal ideation and attempts were assessed by the Composite International Diagnostic Interview Module. Logistic regression analysis was used to evaluate the significance of the association between the dependent and independent variables. Variables with a p-value <0.25 were candidates for the multivariable logistic regression so that predictors of suicidal ideation and attempt were identified at a p-value < 0.05, with a 95% confidence interval (CI), in the final model.

**Results:**

This study reported that 24% and 10.7% of the patients with cancer had suicide ideation and attempted suicide in the previous 12 months, respectively. Being female [adjusted odds ratio (AOR) = 5.35; 95% CI, 2.48–11.54] and having anxiety (AOR = 4.09; 95% CI, 1.85–9.03), psychological distress (AOR = 4.19, 95% CI, 1.61–10.87), and stage IV cancer (AOR = 5.81, 95% CI, 1.73–19.51) were significantly associated with suicidal ideation while having depression [AOR = 3.25, 95% CI, 1.05- 10.06] and anxiety [AOR = 3.50, 95% CI, 1.19-10.32] were significantly associated with attempting suicide.

**Conclusion:**

Nearly one-quarter and one-tenth of the patients with cancer had suicide ideation and attempted suicide in the previous 12 months, respectively. Being female, advanced cancer stage, anxiety, and psychological distress were statistically significantly associated with suicidal ideation. Anxiety and depression were statistically associated with attempting suicide. It is important that oncology professionals routinely perform patient suicidal risk assessment. Consultation services need to be strengthened with psychiatric professionals in cancer treatment centers.

## Background

Suicide is the fatal act of terminating one’s own life and it is a complex process that involves a series of pathways and mechanisms from the initiation of ideation, to planning, and finally to attempting suicide. Suicidal ideation is a critical stage in the suicide process that comes before attempting suicide; it is the main risk factor for death by suicide and can be lethal. The frequency of suicide attempts may be up to 20 times higher than the suicide death rate (1).

One person dies by suicide every 40 seconds, which is a major problem because it abruptly ends a person’s life. Approximately 800,000 suicides have been reported worldwide, and 78% of all completed suicides globally occur in low- and middle-income countries with an estimated global burden of approximately 1.4% (2). The prevalence of suicide in African regions ranges from 0.5% to 1.9% (2). According to another study in the majority of sub-Saharan African countries, the annual suicide fatality rate is projected to be 34,000. Men are three times more likely than women to commit suicide (3). In Ethiopia, there were 7.7 suicide deaths per 100,000 people per year (4).

Cancer is the leading cause of morbidity and the third-leading cause of mortality with 18.1 million new cancer cases and 9.6 million deaths worldwide (5). Cancer has extensive effects on a person’s physical, emotional, and spiritual health (6, 7). Intense feelings such as sadness, depression, fear of disability, pain, and even suicidal feelings may occur at the time of disclosure of the diagnosis and treatment options (6). Suicidal thoughts in patients with cancer can be due to psychological reactions during the cancer diagnosis, the long duration of treatment, repeated hospitalizations, diminished quality of life, limited physical activities, and immunological disturbances (8, 9).Treatment-related factors such as side effects of chemotherapeutic agents and radiation can also contribute to the development of depression and suicidal thoughts (10). To the best of our knowledge, there have only been two psychological autopsy studies investigating cancer patient suicide victims. They suggested that a number of physical, psychological, and existential distresses, such as pain, impairment of physical functioning, depression, loss of independence, and loss of autonomy, contribute to cancer patients’ suicidal thoughts (11, 12).

Due to lifestyle factors, such as smoking (13), work-related risk factors (14), and dietary risks, the burden of cancer is higher in low- and middle-income countries (13). Ethiopia has an increasing population that is more than 110 million people, with a projected parallel increase in the cancer burden (15).

Outside of mental health or psychiatric settings, little is known about suicidality despite the fact that it is frequent in industrialized and developing nations with ongoing medical issues (16). In Ethiopia, suicidal ideation has a higher magnitude among patients with severe mental illness and other chronic illnesses. However, little attention has been paid to identifying suicidal ideation in cancer patients, which is a common chronic problem. Taking into account the issue, the current study was intended to assess the magnitude of and associated factors with suicidal ideation and attempted suicide among patients with cancer at Jimma University Medical Center (JUMC) to support the integration of mental health services with cancer management.

## Methods

### Study period and setting

JUMC is the only teaching and referral hospital in south-western Ethiopia and it is one of the oldest public hospitals in Ethiopia. It is located in Jimma, which is approximately 355 km southwest of the capital city, Addis Ababa. The hospital provides services to 15 million people. The medical center has 1,600 staff in different disciplines. It provides an inpatient service with 32 intensive care units and 800 beds. Approximately 3,260 cancer patients receive an outpatient service from this medical center. The study was conducted from 1 to 30 November 2023.

### Study design

A hospital-based cross-sectional study was conducted at the Cancer Center at the Jimma University Medical Center.

### Source population

All adult patients with cancer treated at the Cancer Centre at the Jimma University Medical Center were the source population.

### Study population

All the patients with cancer who were on regular follow-up at the Cancer Center at the Jimma Medical Center who came to the center during the data collection period were selected to participate in the study.

### Eligibility criteria

#### Inclusion criteria

All cancer patients who were 18 years old or above and attending the Cancer Center at the Jimma Medical Center were included in the study.

#### Exclusion criteria

Those who were seriously ill and unable to communicate were excluded.

### Sample size determination

The sample size was determined using the single population proportion formula, taking a 20% prevalence of suicidal ideation among cancer patients (17) with the following assumptions: 95% CI and 5% margin of error.


$$\text{n}=\frac{{\left(\frac{\text{za}}{2}\right)}^{2}*\text{p}(1-\text{p})}{{\text{d}}^{2}}$$

$$\frac{n\;={\left(1.96\right)}^{2}*0.2*0.8}{0.005^{2}}$$

n=246

By adding a 10% non-response rate, the final sample size was 271.

### Sampling techniques

The consecutive sampling technique was used to select the study participants.

### Study variables

#### Dependent variables

The dependent variables were suicidal ideation and attempted suicide.

#### Independent variables

Socio-demographic Factors: age, sex, religion, ethnicity, marital status, educational status, occupational status, residence, living arrangement, and income.

Psychosocial factors: social support, depression and anxiety, and psychological distress.

Clinical factors: duration of diagnosis, clinical cancer stages, cancer site, comorbidity, cancer sites, type of treatment, pain, family history of suicidal behavior, and a history of suicide.

Substance use: alcohol, tobacco, khat, and other drugs.

### Data collection methods and instruments

A structured questionnaire including sociodemographic characteristics, experience of suicidal ideation and attempted suicide, depression and anxiety, pain, social support, psychological distress, and substance-related factors was used to collect data using face-to-face interviews in the KoboToolbox mobile application. The patient’s chart was also reviewed. Two BSc Psychiatry professionals and one MSc student collected the data after training them for one day about the objective, data collection techniques, maintaining confidentiality, data quality, and techniques of an interview.

### Suicidal ideation and attempted suicide

Suicidal ideation and attempted suicide were assessed using the suicidality module of the World Mental Health (WMH) survey initiative version of the World Health Organization (WHO) Composite International Diagnostic Interview (CIDI). It contains a module that assesses the lifetime, previous 12-month, and 1-month occurrence of suicidal behaviors (suicidal ideation, attempt, and plan). It also assesses the methods used to attempt suicide, the patient’s reason for attempting suicide, and their response after the attempt. Its Amharic version is validated in Ethiopia in both clinical and community settings with internal consistency (Cronbach’s alpha=0.97) (18).

### Alcohol, Smoking, and Substance Involvement Screening Tool

The Alcohol, Smoking, and Substance Involvement Screening Tool (ASSIST) has eight items and is used to screen substance use disorder. The score ranges from 0 to 39. For alcohol, the cut-off score is ≥ 9.5 with 95% sensitivity and 84% specificity, and for tobacco, the cut-off score is ≥ 4 with 97% sensitivity and 62% specificity, whereas for amphetamines (khat), the cut-off score is ≥ 3 with 99% sensitivity and 98% specificity (19).

### Social support

Social support was assessed using the 3-Item Oslo Social Support Scale and scores between 3 and 8 = poor social support, 9–11 = intermediate social support, and 12–14 = strong social support with Cronbach’s alpha of 0.60 (20).

### Hospital Anxiety and Depression Scale questionnaire

The Hospital Anxiety and Depression Scale (HADS) was used to assess depression and anxiety. HADS is a 14-item tool commonly used to screen symptoms of anxiety and depression among patients on follow-up treatment. The 14-item scale has two subscales for anxiety (HAD-A) and depression (HAD-D) and each contains 7 items. The score ranges from 0–21 (0–7 = normal, 8–10 = borderline abnormal, and 11–21 = abnormal). HADS is validated in Ethiopia with a reported internal consistency of 0.78 for HAD-A, 0.76 for HAD-D, and 0.87 for full scale (21).

### Numerical Pain Rating Scale

The Numerical Pain Rating Scale (NRS) includes horizontal line of 10 units with the two ends indicating the extremes of pain. The participants were asked to place a mark indicating where the current pain lies on the line. The left limit usually represents none or no pain, whereas the right one usually represents the worst possible pain. To use the scale, the values on the pain scale correspond to pain levels as follows: 0 = no pain, 1–3 = mild pain, 4–6 = moderate pain, and 7–10 = severe pain (22).

### Questionnaire on Distress in Cancer Patients

The Questionnaire on Stress in Cancer Patients (QSC-R10) is a 10-item screening instrument for self-assessment of psychosocial distress in cancer patients. It is a valid and reliable questionnaire to detect distress in cancer patients with high acceptance among professionals and patients with high reliability (Cronbach’s alpha = 0.85) and test-retest reliability (ICC = 0.89). A cut-off score of >14 demonstrated good sensitivity (81.0%) and specificity (73.2%) and is suitable to determine the need for psychosocial support (23).

### Data quality control

To assure data quality, the questionnaire was translated into the local languages (Amharic language and Afan Oromo) for data collection and back-translated to English to check its consistency. The questionnaire was pre-tested 1 week before the actual data collection on 5% (14) of the patients at JUMC and was not included in the main study. Two BSc Psychiatry professionals and one MSc student collected the data after 1 day of training them about the objective, data collection techniques, maintaining confidentiality, data quality and techniques of an interview. The data collectors were supervised daily and the field questionnaires were checked daily by the supervisors and principal investigator and any problem was solved by discussion with the supervisor, the investigator, and data collectors.

### Data processing and analysis

The data was cleaned, coded, and checked for any missing information after being exported from KoboToolbox to SPSS for analysis. Descriptive statistics are presented by frequency tables, charts, graphs, and text.

Bivariate logistic regression analysis was used to ascertain the significance of the association between the dependent and independent variables. Candidate variables for the final model (multivariable binary logistic regression) were identified with a p-value < 0.25. Variables that had a significant association with the outcome variable in the binary logistic regression analysis were introduced into the multi-variable binary logistic regression and finally, predictors of suicidal ideation and attempted suicide were identified with a p-value < 0.05, 95% CI. Hosmer–Lemeshow goodness and maximum likelihood were checked for model fitness and variance inflation factors (VIFs) were used to check relationships between the variables.

### Operational definitions

Suicidal ideation was defined if the respondent answered the question, “Have you seriously thought about committing suicide within the last 12 months?” with “Yes” (24).

A suicide attempt was defined if the respondent answered the question, “Have you attempted committing suicide in the past 12 months?” with “Yes” (24).

Depression was defined as individuals who scored ≥8 on the depression 7-item subscale in the HADS (21).

Anxiety was defined as individuals who scored ≥8 on the anxiety 7-item subscale in the HADS (21).

Social support was assessed by the 3-item Oslo Social Support Scale. The sum score scale ranges from 3 to 14 and has three categories: poor social support (3–8), moderate support (9–11), and strong social support (12–14) (25).

NRS: the pain levels were calculated as follows: 0 = no pain,1–3 = mild pain, 4–6 =moderate pain, and 7–10 score = severe pain (26).

Stress in Cancer Patients (QSC-R10): A cut-off score of >14 of the 10-item screening instrument was used to declare the presence of distress in the cancer patients (23).

## Results

### Sociodemographic characteristics of participants

A total of 271 respondents were enrolled in the study with a 100% response rate. The mean and median ages of participants were 45.24 ± 14.11 and 45 years respectively with an inter quartile range (IQR) of 34–56 years. Of the total participants, 51.3% were women. With respect to the educational and occupational status of the respondents, 96 (35.4%) attended secondary education and 125 (46.1%) were employed. Regarding the residential setting, 138 (50.9%) were from rural areas. Approximately half (125, 46.1%) of the respondents were married (**Table 1**).

**Table 1:** Sociodemographic characteristics of the patients with cancer at Jimma University Medical Center, Jimma, Ethiopia, 2023 (n=271).

Variable	Category	Frequency	Percentage
Age	17–30	49	18.1
	31–45	90	33.2
	>45	192	48.7
Sex	Male	132	48.7
	Female	139	51.3
Marital status	Single	49	18.1
	Married	125	46.1
	Divorced	35	12.9
	Separated	29	10.7
	Widowed	33	12.2
Educational status	No formal education	96	35.4
	Primary education	53	19.6
	Secondary education	67	24.7
	College and above	55	20.3
Occupational status	Employed	125	46.1
	Unemployed	56	20.7
	Farmer	39	14.4
	Merchant	26	9.6
	Student	22	8.1
	Others*	3	1.1
Living arrangement	Living with family	230	84.9
	Living alone	41	15.1

Others*: houseworker, retirement.

### Clinical and psychosocial characteristics of the respondents

A majority (139, 51.3%) of participants were 0–5 months since being diagnosed with cancer. Breast (67, 24.7%) and cervical (57, 21.4%) were the most common cancer types. More than one-third of participants were diagnosed with stage III cancer (105, 38.7%). In total, 113 (41.7%) of the respondents had moderate pain. Nearly half (128, 47.2%) of the participants had poor social support. Of the total study participants, 172 (63.5%), 149 (55%), and 181 (66.8%) were found to have depression, anxiety, and stress symptoms, respectively. More than half (140, 51.7%) of the participants received chemotherapy as their treatment modality. Regarding substance use, 94 (34.7%) of participants had ever used substances and 34 (14%) were currently using substances (**Table 2**).

**Table 2:** Description of clinical, psychosocial, and substance use characteristics of patients with cancer at Jimma University Medical Center, Jimma, Ethiopia, 2023 (n = 271).

Variable	Category	Frequency	%
Time since diagnosis	0–5	139	51.3
	6–11	81	29.9
	>12	51	18.8
Current cancer stage	Stage I	46	17
	Stage II	87	32.1
	Stage III	105	38.7
	Stage IV	33	12.2
Type of treatments	Chemotherapy	140	51.7
	Radiotherapy	70	25.8
	Surgery	33	12.2
	Chemotherapy and radiotherapy	12	4.4
	Palliative care	10	3.7
	Other	6	2.2
Family history of suicidal behavior	Yes	47	17.3
	No	224	82.7
Previous history of suicidal behavior	Yes	36	13.3
	No	235	86.7
Any comorbidity	Yes	61	22.5
	No	210	77.5
Presence of pain	Mild	54	19.9
	Moderate	113	41.7
	Severe	104	38.4
Social support	Poor social support	128	47.2
	Intermediate social support	112	41.3
	Strong social support	31	11.5
Depressive symptoms	Yes	172	63.5
	No	99	36.5
Anxiety symptoms	Yes	149	55
	No	122	45
Psychological distress	Yes	181	66.8
	No	90	33.2
Ever substance use	Yes	94	34.7
	No	177	65.3
Current substance use	Yes	38	14
	No	233	86
Cancer type	Breast	67	24.7
	Cervical	58	21.4
	Head	38	14.1
	Neck	31	11.4
	Lung	20	7.3
	Liver	19	7.1
	Prostate	16	5.9
	Blood	13	4.8
	Others*	9	3.3

Others*: pancreatic cancer, sarcoma, skin cancer.

### Prevalence of suicidal ideation and attempted suicide among cancer patients

This study found that the 12-month prevalence of suicidal ideation and attempted suicide was 65 (24%) with a 95% CI of 19–29, and 29 (10.7%) with a 95% of CI 7–14, respectively.

Of the total number of participants, 28 (58.3%), 12 (25%), and 8 (16.7%) had attempted suicide once, twice, and more than two times, respectively. Of these suicide attempters, 18 (37.5%) reported that their suicide attempt was related to their physical illness and 32 (66.7%) made serious attempts. The most common method of attempt they used was hanging (27, 58.7%) (**Table 3**).

**Table 3:** Prevalence of suicidal ideation and attempted suicide among patients with cancer at Jimma University Medical Center, 2023 (n=271).

Variable	Category	Frequency	%
Lifetime suicidal ideation	Yes	78	28.8
	No	193	71.2
1-year suicidal ideation	Yes	65	24
	No	206	76
1-month suicidal ideation	Yes	40	14.8
	No	231	85.2
Lifetime suicide attempt	Yes	48	17.7
	No	223	82.3
1-year suicide attempt	Yes	29	10.7
	No	242	89.3
1-month suicide attempt	Yes	9	3.3
	No	262	96.7
A lifetime suicidal plan	Yes	37	12.5
	No	237	87.5
1-year suicidal plan	Yes	25	9.2
	No	246	90.8
1-month suicidal plan	Yes	20	7.4
	No	251	92.6
Frequency of suicide attempts	Once	28	58.3
	Two times	12	28
	More than two times	8	16.7
Reasons for suicide attempt	Physical illness	18	37.5
	Poverty	2	4.2
	Death in family	3	6.2
	Financial loss	5	10.4
	Mental illness/problem	4	8.4
	Family conflict	2	4.2
	Family conflict and physical illness	5	10.4
	Financial loss and physical illness	6	12.5
	Family conflict, financial loss, and physical illness	3	6.2
Method of suicidal attempt	Hanging	27	58.7
	Use sharp tools	10	21.7
	Poisoning	7	15.2
	Others	2	4.4
Reasons that describe the patient’s response	Made serious attempt	32	66.7
	Methods used were not effective/foolish	10	20.8
	Attempt was made to seek help	6	12.5

Others: jumping from high place, drowning.

### Factors associated with suicidal ideation

In the bi-variable logistic regression analysis, variables including sex, marital status, educational status, living arrangement, family history of suicide, cancer stage, depressive symptoms, anxiety symptoms, pain, substance use, and psychological distress symptoms were candidates for multiple logistic regression with p-values < 0.25. However, in the multivariate logistic regression analysis, variables including being female, advanced cancer stage, anxiety symptoms, and psychosocial distress were statistically significantly associated with suicidal ideation with p-values ≤ 0.05.

In this study, the odds of having suicidal ideation among the female respondents were approximately 5.35 times higher as compared to male participants [adjusted odds ratio (AOR) = 5.35; 95% CI = 2.48–11,54]. The odds of having suicidal ideation among respondents who had anxiety symptoms and psychosocial distress were 4.09 and 4.19 times higher as compared to respondents who had no anxiety symptoms and psychosocial distress, respectively [(AOR = 4.09; 95% CI 1.85–9.03) and (AOR = 4.19, 95% CI 1.61–10.87)] and the odds of having suicidal ideation among participants who had an advanced stage of cancer was 5.81 times higher as compared to participants with earlier stages of cancer (AOR = 5.81; 95% CI 1.73–19.51) (**Table 4**).

**Table 4:** Bivariable and Multivariable regression analysis of suicidal ideation among patients with cancer visiting Jimma University Medical Center, Jimma, Ethiopia, 2023 (n=271).

Variable	Category	Suicidal ideation		COR (95% CI)	AOR (95% CI)
		Yes	No		
Sex	Male	17	115	1	1
	Female	48	91	3.57 (1.92, 6.62)	5.35 (2.48,11.54)**
Educational status	No formal	17	79	1	1
	Primary education	14	39	.41 (.19, .87)	0.91 (0.32, 2.60)
	Secondary education	15	52	.68 (.30, 1.55)	1.49 (0.54, 4.10)
	College and above	19	36	.55 (.25, 2.22)	1.55 (0.57, 4.20)
Marital status	Married	25	100	1	1
	Single	14	35	1.60 (.75, 3.42)	1.47 (0.55, 3.98)
	Divorced/ Separated	16	48	1.33 (.65, 2.73)	1.04 (0.39, 2.81)
	Widowed	10	23	1.74 (.73, 4.12)	1.27 (0.41, 4.00)
Living arrangement	Living with families	47	183	1	1
	Living alone	18	23	3.05 (1.52, 6.11)	1.67 (0.65, 4.28)
cancer stage	Stage I	10	36	1	1
	Stage II	16	71	.81 (.33, 1.97)	1.40 (.49, 4.05)
	Stage III	24	81	1.07 (.46, 2.46)	1.44 (.52, 3.96)
	Stage IV	15	18	3.00 (1.13, 7.99)	5.81 (1.73,19.51)
Psychological distress	No	8	82	1	1
	Yes	57	124	4.71 (2.14, 10.39)	4.19 (1.61,10.87)**
Pain	Mild pain	16	38	1	1
	Moderate pain	18	95	0.45 (.21, .97)	0.57 (0.22, 1.47)
	Severe pain	31	73	1.01 (.49, 2.07)	0.95 (0.35, 2.57)
Family history of suicide	No	50	174	1	1
	Yes	15	32	1.63 (.82, 3.25)	1.52 (0.62, 3.72)
Depression	No	14	85	1	1
	Yes	51	121	2.56 (1.33, 4.92)	1.84 (0.83, 4.05)**
Anxiety symptoms	No	14	108	1	1
	Yes	51	98	4.02 (2.09, 7.70)	4.09 (1.85, 9.03)**
Ever substance use	No	38	139	1	1
	Yes	27	67	1.47 (.83, 2.61)	1.68 (0.80, 3.50)

1=Reference group; **p<0.01; COR, crude odds ratio; AOR, adjusted odds ratio.

### Factors associated with attempted suicide

In the bi-variable logistic regression analysis, variables including marital status, living arrangement, previous suicidal behavior, anxiety and depressive symptoms, psychological distress, date of diagnosis confirmation, and severe pain were candidates for the multiple logistic regression with p-values < 0.25. However, in the multivariate logistic regression analysis, anxiety and depression symptoms were statistically associated with attempted suicide with p-values less than 0.05.

In this study, the odds of having attempted suicide among the respondents who had anxiety symptoms were 3.50 times higher as compared to respondents who had no anxiety symptoms (AOR = 3.50; 95% CI 1.19–10.32), and those with depressive symptoms were 3.25 times more likely compared to respondents without depressive symptoms (AOR = 3.25; 95% CI 1.05–10.06) (**Table 5**).

**Table 5:** Bivariable and multivariable regression analysis of suicidal attempts among patients with cancer visiting Jimma University Medical Center, Jimma, Ethiopia, 2023 (N=271).

Variable	Category	Suicide attempt		COR (95% CI)	AOR (95% CI)
		Yes	No		
Marital status	Married	7	118	1	
	Single	6	43	2.35 (.75, 7.39)	0.42 (0.11, 1.55)
	Divorced/ Separated	10	54	3.12 (1.13, 8.64)	0.85 (0.22, 3.26)
	Widowed	6	27	3.75 (1.17, 12.04)	0.99 (0.29, 3.40)
Living arrangement	Living with family	19	211	1	1
	Living alone	10	31	3.58 (1.53, 8.41)	0.73 (0.26, 2.01)
Pain	Mild pain	5	49	1	
	Moderate pain	6	107	.550 (.160, 1.888)	0.81 (0.24, 2.73)
	Severe pain	18	86	2.051 (.717,5.868)	0.34 (0.11, 1.02)
Previous history of suicide	No	7	213	1	
	Yes	29	22	2.337 (.918, 5.952)	0.63 (0.22, 1.80)
Psychological distress	No	5	85	1	1
	Yes	24	157	2.599 (.957,7.057)	0.78 (0.26, 2.39)
Anxiety symptoms	No	5	117	1	1
	Yes	24	125	4.493 (1.660, 112.163)	3.50 (1.19,10.32)**
Depression symptoms	No	5	94	1	1
	Yes	24	148	3.049 (1.124, 8.267)	3.25 (1.05,10.06)**
Date of cancer confirmation	0–5 months	14	126	.597 (.234, 1.521)	0.75 (0.24, 2.33)
	6–11months	7	73	.515 (.175, 1.521)	0.41 (0.12, 1.42)
	>=12 months	8	43	1	

1=Reference group; **p<0.01; COR, crude odds ratio; AOR, adjusted odds ratio.

## Discussion

In this study, the magnitude of suicidal ideation and attempted suicide in the previous 12 months was found to be 24% (95% CI: 19–29) and 10.7% (95% CI: 7–14), respectively. The magnitude of suicidal ideation in this study was consistent with studies conducted in Spain, 25.24% (27); China, 21.8% (28); South Korea, 24.74% (29); Italy, 20% (30); Egypt, 28.55% (31); and Mekelle, 27.9% (32).

However, the magnitude of suicidal ideation in this study was lower than the studies conducted in Portugal, with 34.6% (33), and South Africa, with 71.4% (34). A possible reason for this discrepancy could be differences in sample size. Furthermore, in the Portuguese study, the tool used to assess suicidal ideation was the Beck Scale for Suicide Ideation and the study focused exclusively on patients with advanced stages of disease and specific cancers found in four sites (lung, breast, cervical, and head and neck), while our study included various types of cancer. Our study was also different from the South African study as it only focused on participants with cervical cancer.

The finding of this study was also higher than studies conducted in the USA, 12.8% (35), and South Korea, 12.7% (29). The study conducted in the USA included adult survivors of childhood cancer and used the Beck Scale for Suicide Ideation as the tool, which could have contributed to this discrepancy. Furthermore, the result of the study conducted in South Korea was different from the current study which might be explained the study participants, since it only incorporated patients with advanced types of cancer.

The prevalence of suicide attempts in this study was 10.7% (95% CI 7–14) which was in agreement with studies conducted in Tunisia and Mekelle, with 7.3% (36) and 8.4% (36), respectively. However, the prevalence of suicide attempts in this study was higher than in studies conducted in Sweden, with 1.7% (37), and Colombia, with 4.5% (38). A possible explanation for these differences may be differences in study design, study participants, and sociocultural differences across countries. For example, the study conducted in Sweden was a population-based cohort study among adolescents and young adults after receiving a cancer diagnosis and it used a retrospective study design by reviewing inpatient participants.

The study in Colombia used the Scale of Suicidal Ideation (SSI), the 9-item Beck Depression Scale (BDI), and the Beck Hopelessness Inventory (BHS), which may have contributed to these discrepancies. Furthermore, the finding of this study was lower than a study conducted in China that reported the prevalence of suicide attempts to be 14.6% (39). A possible reason for this discrepancy could be that the study done in China only recruited patients with an advanced stage of cancer, while the current study recruited all stages. This may result in an increase in the magnitude of suicidal attempts since suicidal behavior is more common among those in the advanced stages of the disease than early stages due to patients being at high risk for psychological distress (40).

Regarding the factors associated with suicidal ideation, being female was significantly associated with suicidal ideation among cancer patients according to this study. This finding was consistent with studies conducted in South Korea (29), Portugal (33), Gondar (41), and Mekelle (17). The implication for this might be that women are more vulnerable to psychosocial stressors and more likely to have depression compared to men; different cultural and religious influences may also play a role. This might lead them to be depressed and in the end, think about ending their lives (27, 42). However, this study contradicts systematic review and meta-analysis studies done across different countries and settings (43, 44). A number of reasons could be responsible for the differences between this study and earlier systematic reviews and meta-analyses carried out in various nations and environments. Different outcomes could emerge from variations in study demographics, sample sizes, and methodologies. Cultural, socioeconomic, and healthcare system setting variations may have an impact on the results and generalizability of the research. Furthermore, disparities in the statistical methods, data collection strategies, and measurement instruments employed in various investigations may also be a factor. One possible explanation for the observed discrepancies is publication bias, which occurs when only noteworthy findings are reported. Furthermore, contextual variations including how policies are applied, how easily healthcare is accessible, and how society views the issue under study may lead to different results in different contexts. In conclusion, systematic reviews and meta-analyses make more robust conclusions in different aspects than single studies.

In this study, having anxiety symptoms was also found to be associated with suicidal ideation. This agrees with studies conducted in China (39), South Korea (29), and Mekelle (36). The reason for this was that anxiety symptoms are explained by an excessive feeling of worry and anxiety that is beyond the individual’s control. It overwhelms the thoughts of the individual and can lead individuals to suicidal ideation (45). Another explanation could be that anxiety, by altering the brain chemistry, particularly affecting serotonin neurotransmitters, plays a significant role in mood regulation, thereby increasing the risk of suicidal ideation (2).

This study also reported that suicidal ideation was associated with advanced stages of cancer compared to early stages. This was supported by studies conducted in China (39), Mekelle (36), and Gondar (41). This could be due to increased suffering from pain and loss of physical appearance due to anticancer drug side effects and surgery. Another possible reason may be that anticancer drugs have depressant effects on the patients and depression may increase the risk of suicide (36).

The other factor significantly associated with suicidal ideation was psychological distress. This finding was supported by studies conducted in Germany (45) and Canada (46). This could imply that the more distress a patient experiences, the more frequent are suicidal thoughts. It could also be explained by the hopelessness, loss of meaning, and demoralization caused by the psychological distress (38).

Regarding factors associated with suicide attempts, having anxiety symptoms was found to be significantly associated with suicide attempts. This was consistent with a study conducted in South Korea (47). The reason for this could be that an increase in the level of anxiety among cancer patients reduced their spiritual wellbeing, which has a protective function during stressful situations and results in negative outcomes such as suicide attempts (48).

The other factor significantly associated with suicide attempts was depression. This was supported by studies conducted in the USA (37) and Gondar (41).The possible reason for this could be that depression causes the patients to have negative beliefs about themselves, the environment, and the world that lead them to attempt to end their lives. Another possible explanation could be that depressed individuals feel hopeless and have a loss of interest; the cardinal features of depression that lead them to attempt suicide (49, 50).

### Strengths and limitations of the study

A strength of the study was that it used a sufficient sample to represent the target population and used standard and validated tools to assess both the dependent and independent variables. However, data collection used the interview method, which may result in a recall problem for some symptoms and social desirability bias. In this study, only adult cancer patients were included, so it is difficult to generalize the results to all cancer patients. In addition, the study used a cross-sectional design, which hindered the accurate examination of the causal relationship between suicidal behaviors and risk factors, and also employed a non-probability sampling method, which makes it difficult to make generalizations. Another limitation of this study was the potential difficulty in distinguishing between psychiatric symptoms directly attributed to cancer treatments and those arising from the cancer diagnosis or other psychosocial factors, which could be better addressed through a longitudinal design and the incorporation of qualitative data from patient interviews.

## Conclusions

According to this study, the prevalence of suicidal ideation and attempted suicide among patients with cancer was high. This study reported that being female, advanced cancer stage, anxiety symptoms, and psychosocial distress had a statistically significant association with suicidal ideation. Anxiety and depression symptoms were statistically associated with attempted suicide. To address the limitations of this study, future research should employ a longitudinal design to monitor changes in psychiatric symptoms over time and clearly differentiate between symptoms resulting from treatments versus those related to the cancer diagnosis or psychosocial factors, while also incorporating qualitative data from patient interviews to gain deeper insights into their experiences and perceptions.

## Data Availability

The raw data supporting the conclusions of this article will be made available by the authors, without undue reservation.
